# U_4_ at the 3′ UTR of PB1 Segment of H5N1 Influenza Virus Promotes RNA Polymerase Activity and Contributes to Viral Pathogenicity

**DOI:** 10.1371/journal.pone.0093366

**Published:** 2014-03-27

**Authors:** Wei Sun, Jing Li, Pengfei Han, Yinhui Yang, Xiaoping Kang, Yuchang Li, Jiaming Li, Yu Zhang, Xiaoyan Wu, Tao Jiang, Chengfeng Qin, Yi Hu, Qingyu Zhu

**Affiliations:** State Key Laboratory of Pathogens and Biosecurity, Beijing Institute of Microbiology and Epidemiology, Beijing, PR China; University of Edinburgh, United Kingdom

## Abstract

The viral RNA-dependent RNA polymerase has been found to contribute to efficient replication in mammalian systems and to the high pathogenicity of H5N1 influenza A virus in humans and other mammals. The terminal untranslated regions of the viral segments perform functions such as polyadenylation and contain signals for genomic packaging and initiation of RNA synthesis. These sequences are highly conserved, apart from a U/C polymorphism at position 4 of the 3′ end, most often seen in the polymerase gene segments. However, no study has yet tested whether the untranslated regions of H5N1 make any contribution to its high pathogenicity. Herein, the association of the fourth nucleotide at the 3′ end of the untranslated region in segment 2 (PB1), of A/Vietnam/1194/2004 (H5N1), with pathogenicity was examined in mice. To this end, an RNA polymerase reporter system was constructed, and viruses with mutations at this site were rescued. Results showed the U_4_ in PB1 was found to contribute to greater amounts of RNA-dependent RNA polymerase activity and differentially regulate genomic transcription and replication. Although a recombinant H5N1 virus with the rarer C_4_ sequence in all eight segments was viable and replicated to high titers in vitro, replacing a single U_4_ at the 3′ termini of the PB1 gene segment enhanced viral reproduction and more pathogenesis. In this way, these data showed the importance of untranslated regions of H5N1 influenza virus to pathogenicity.

## Introduction

The genome of the influenza A virus (IAV) is composed of eight single-stranded RNA segments of negative polarity. The eight viral RNA (vRNA) segments are transcribed and replicated by the viral RNA-dependent RNA polymerase (RdRp) which is assembled by PB1, PB2, and PA, in association with nucleoprotein (NP) in the host cells [1], [2]. During a replication cycle, vRNAs are transcribed into mRNA and copied into cRNA molecules, which in turn generate more vRNAs as templates [3], [4]. Each genomic segment of IAV contains coding regions and untranslated regions (UTRs). UTRs comprise universally conversed sequences and segment-dependent sequences at both the 5′ and 3′ ends. And the first 13 nt at the 5′ end and the first 12 nt at the 3′ end are highly conserved and segment-independent [5]. These nucleotides are partially complementary and can form duplex structures, such as panhandle structures [6], which can be recognized by RdRp to initiate transcription and replication [7].

UTRs of IAVs, believed to perform many functions, contain signals for polyadenylation, genomic packaging and RNA synthesis. Deletions or replacement of the NA UTRs with UTRs of PB1 or NS can decrease the NA vRNA in infected cells and in virions [8]. Down-regulation of NS vRNA incorporation into virions, which can be caused by deletion of the 3′ UTR from the NS gene, also indicated that UTRs could affect the packaging of viral RNA [9]. Only one single natural variation, U or C at position 4 of the 3′ end of the vRNA (U_4_ or C_4_), was observed in the first 12 nt at the 3′ end of any influenza virus. Lee et al. confirmed that the U_4_ and C_4_ in NA of A/WSN/33 may be involved in the regulation of viral transcription and replication [10]. However whether this variation in other gene segments, especially in polymerase-related gene segments, could share the same function was not clear. And whether the variation contributes to viral pathogenicity was also not identified.

The H5N1 influenza virus is currently the most pathogenic to humans of all known influenza viruses. Multiple viral determinants of the high pathogenicity of the virus have been identified in recent years. Hemagglutinin (HA) was found to be one key determinant of virulence. The HA of H5N1 virus targets SA-α2,3-Gal receptor, which located lower respiratory tract and lungs [11], [12], and causes severe pneumonia. Multibasic cleavage sites in HA of H5N1 virus facilitate viral maturation and contribute to pathogenicity [13], [14]. Besides HA segment, the amino acids at positions 627 and 701 of PB2 contribute to the pathogenicity of the virus, and are widely believed to be important for viral replication and host range. Moreover, nonstructural 1 protein (NS1), which has a E92D substitution, has shown significantly attenuated virulence due to viral resistance to antiviral effects of interferons and tumor necrosis factor α [15], [16]. Altogether, the importance of determinants within coding region of viral genomic segments has been widely evaluated and confirmed. However, few studies have focused on the association of UTRs of H5N1 genomic segments with viral virulence.

To determine whether there is any association between UTRs and the high pathogenicity of the H5N1 influenza virus, we determined the role of U_4_/C_4_ variation in the PB1 segment of in A/Vietnam/1194/2004 (H5N1, VN1194) on RNA-dependent RNA polymerase activity, genomic RNA expression, virus reproduction, and pathogenicity.

## Results

### Promotion of U_4_ to transcription activity of RNA-dependent RNA polymerase

**Figure 1:**
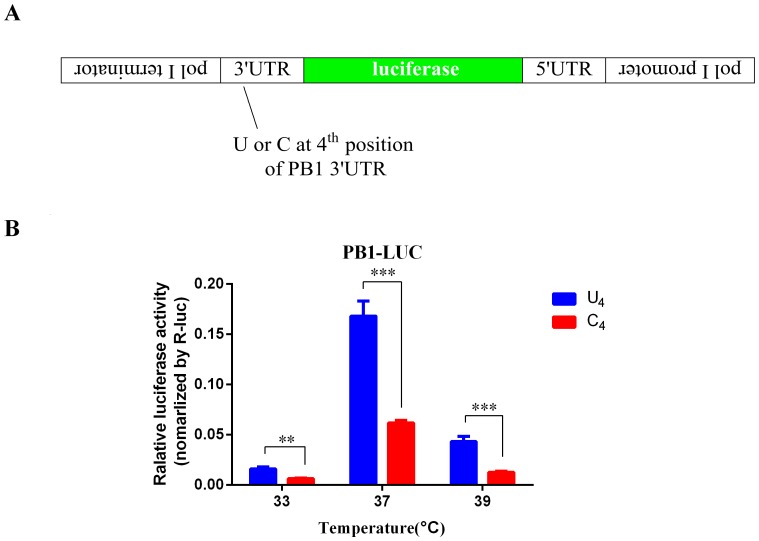
Transcription activity of PB1(U)-LUC and PB1(C)-LUC. (A) Schematic diagram for construction of PB1(U)-LUC and PB1(C)-LUC. The luciferase gene was inserted between 3′ UTR and 5′ UTR as the anti-sense orientation. (B) Transcription activity of PB1(U)-LUC and PB1(C)-LUC was measured as relative luciferase activity, normalized by *Renilla* luciferase activity. The assay was performed in 293T cell lines in 3 replicates. The mean relative activity (±SEM) values are shown. The *P* values for significance for each group were calculated using the Student's t test. (**) *P*<0.01; (***) *P*<0.001.

A previously reported strategy was used to evaluate the transcription activity of RNA-dependent RNA polymerase (RdRp) *in vitro* [17]. Artificial RNA segments encoding firefly luciferase under control of the PB1 UTR of A/Vietnam/1194/2004(H5N1), named as PB1(U_4_)-LUC and PB1(C_4_)-LUC were constructed (as shown in Fig. 1A). A dual-luciferase assay was performed using transfection with 6 plasmids and measured both Firefly and *Renilla* luciferase activity. The significant difference in RdRp activity contributed by PB1(U_4_) and PB1(C_4_) was confirmed post analysis of relative luciferase activity of PB1(U_4_)-LUC and PB1(C_4_)-LUC reporter (as showed in Fig. 1B): the transcription activity of PB1(C_4_)-LUC for 37°C was 36.9±2.4% of that of PB1(U_4_)-LUC, indicating that U_4_ confers to the greater advantage of transcription (*P*<0.01). Similar statistically significant results were found at 33°C (40.6%±3.5%) and 39°C (29.7%±3.5%). These results indicate that present of U_4_ promotes greater transcription activity of RdRp across a range of temperatures.

### Discriminate regulation of PB1 mRNA, cRNA and vRNA by U4

**Figure 2:**
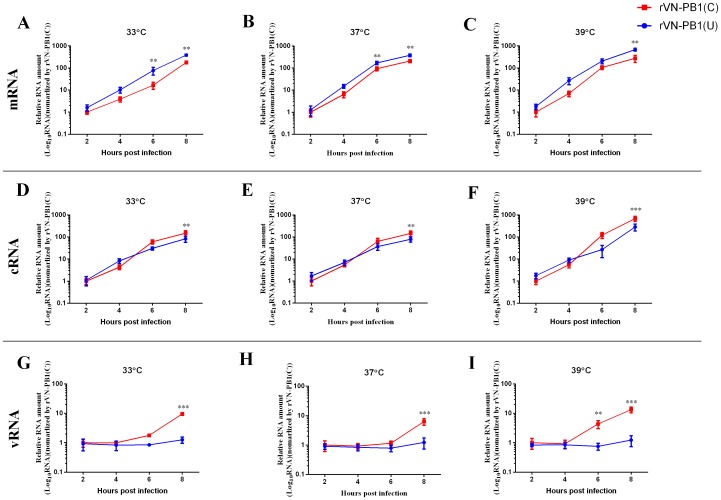
mRNA, cRNA and vRNA expression levels of PB1 gene. The three kinds of RNA relative RNA amounts at 33°C ((A), (D) and (G)), at 37°C (B), (E) and (H) and at 39°C (C), (E) and (I). All data in each figure were analyzed with 2^−ΔΔCt^ method compared with the RNA amounts of rVN-PB1(C) virus at 2 h.p.i.. The mean relative activity and standard deviation (error bar) were calculated from the results of three independent experiments. The data were analyzed with two-way ANOVA using GraphPad Prism Software, and the *P* values were calculated. (*) *P*<0.05; (**) *P*<0.01; (***) *P*<0.001.

To examine the contribution of U_4_ and C_4_ on viral RNA expression, viruses were rescued, either containing C_4_ in all eight segments (rVN-PB1(C)) or containing seven segments with C_4_ and segment 2 with U_4_ (rVN-PB1(U)). MDCK cells were then infected with rVN-PB1(C) or rVN-PB1(U) at MOI of 0.01 at 33°C, 37°C and 39°C. After 2, 4, 6 or 8 hours post infection, the cells were harvested to extract total RNA. Then the mRNA, cRNA and vRNA of PB1 were quantified with Tag-primed real-time RT-PCR assay [18], and the relative RNA amount was calculated using 2^-ΔΔCt^ method and expressed as log_10_2^−ΔΔCt^. As shown in Fig. 2, differences were observed in the patterns of temporal regulation of viral genomic RNA synthesis. At 33, 37 or 39°C, the mRNA copies of both viruses increased time-dependently. More PB1 mRNA was produced by rVN-PB1(U) than rVN-PB1(C), at 8 h.p.i. (Fig. 2A–C) (385.3±40.1 vs 298.4±24.3 at 33°C, 320.7±50.1 vs 238.3±35.7 at 37°C, 680.6±100.5 vs 276.0±99.3 at 39°C), while less cRNA and vRNA of PB1 were synthesized by rVN-PB1(U) than rVN-PB1(U) (Fig. 2D–I). There were significant differences between the two viruses for cRNA (*P*<0.01 for 33°C, *P*<0.01 for 37°C and *P*<0.001 for 39°C, respectively), and vRNA levels at 8 h.p.i. (*P*<0.001 for all three temperatures and *P*<0.05 for 39°C at 6 h.p.i., respectively). These results indicate that the variation of U_4_ and C_4_ could differentially regulate viral transcription and replication in the H5N1 background. Moreover, the similar patterns can also be found at lower temperature (33°C) and higher temperature (39°C), which imply that acquisition of U_4_ from C_4_ could up-regulate transcription and down-regulate replication.

### Contribution of U_4_ on virus reproduction

**Figure 3:**
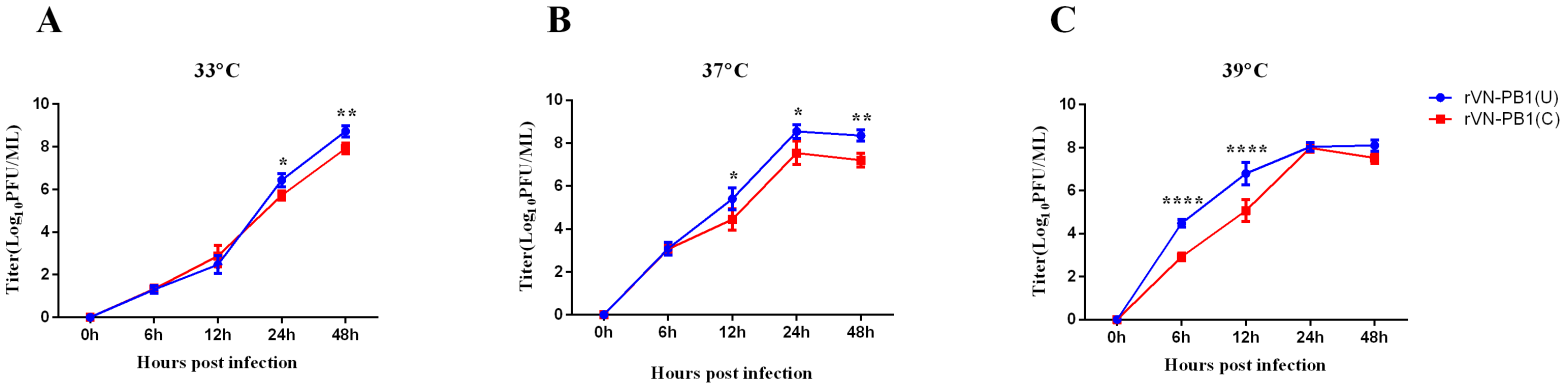
Growth kinetics of viruses with U_4_ and C_4_. (A), (B), and (C) The titers of rVN-PB1(U) and rVN-PB1(C) after infection and at different temperatures. The mean relative activity and standard deviation (error bar) were calculated from the results of three independent experiments. The data were analyzed with two-way ANOVA using GraphPad Prism Software, and the *P* values were calculated. (*) *P*<0.05; (**) *P*<0.01; (***) *P*<0.001; (****) *P*<0.0001.

To determine the characteristics of growth kinetics of rVN-PB1(U) and rVN-PB1(C) viruses, MDCK cells were infected with these viruses at an MOI of 0.01 at 33°C, 37°C or 39°C. At all temperatures, the growth properties of rVN-PB1(U) and rVN-PB1(C) viruses showed somewhat similar patterns. At 33°C both mutant viruses replicated at equal rates and showed similar trends. Both viral titers increased continuously until the detection period ended (Fig. 3A). However, rVN-PB1(U) replicated more efficiently than rVN-PB1(C), and their growth curves reached a plateau at 24 h at 37°C (Fig. 3B), which indicates that U_4_ confers a growth advantage to the virus at 37°C. At 39°C, during the early stage of viral reproduction, the titer of rVN-PB1(U) was found to be significantly higher than that of rVN-PB1(C), but the latter virus caught up at 24 h pi (Fig. 3C). Taking together, these data suggests that U_4_ confers a growth advantage than C_4_ to the H5N1 virus in cells.

### Enhancement of U_4_ to virulence of H5N1 in mice

**Figure 4:**
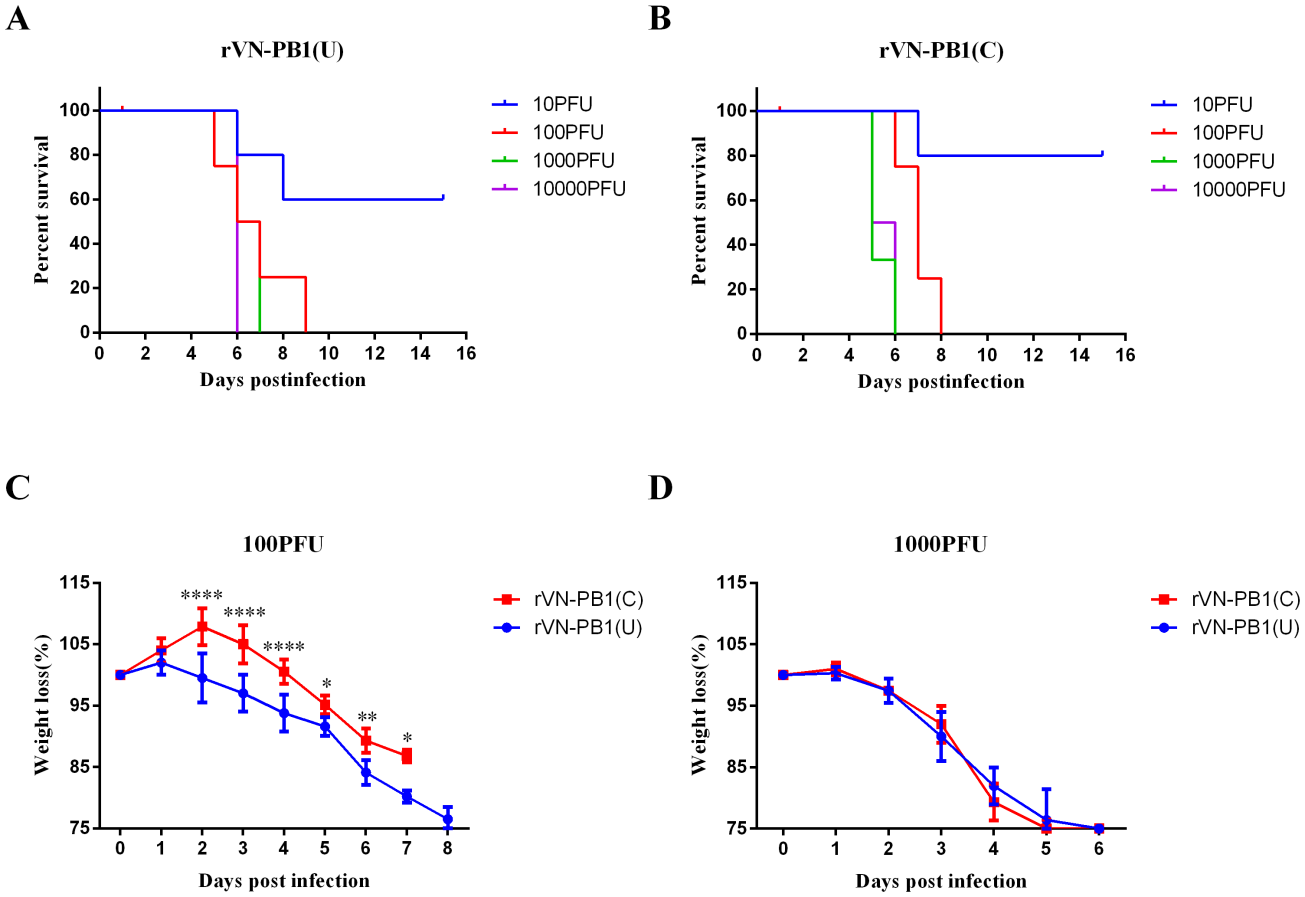
Duration of survival and weight loss in Balb/c mice infected with viruses containing U_4_ and C_4_. The durations of survival of mice infected with rVN-PB1(U) (A) and rVN-PB1(C) (B) with increasing doses of virus were recorded for 15 days. The LD50 titers (median lethal dose, 50% mouse lethal dose) were calculated using the method described by Reed and Muench in 1938 and expressed in PFU. Results showed 13.6 PFU for rVN-PB1(U) and 23.7 PFU for rVN-PB1(C), respectively. (C) and (D) Weight loss of mice infected with rVN-PB1(U) and rVN-PB1(C) at 100 PFU and 1000 PFU. The mean relative activity and standard deviation (error bar) were calculated. The data were analyzed with two-way ANOVA using GraphPad Prism Software, and the *P* values were calculated. (*) *P*<0.05; (**) *P*<0.01; (***) *P*<0.001, (****) *P*<0.0001.

To determine the influence of U_4_/C_4_ variation in PB1 of VN1194 virus on virulence in mice, the mortality and morbidity of rVN-PB1 (U) and rVN-PB1(C) were evaluated in mice. Mice infected with doses higher than 100PFU of rVN-PB1(U) or rVN-PB1(C) virus were dead before day 10 after infection (Fig. 4A and 4B). This indicated the high mortality of both viruses. However, after an intranasal inoculation of 10 PFU, more mice in the rVN-PB1(U) group died than in other groups, and the average duration of survival of mice in the rVN-PB1(U) group was less than that of the mice in the rVN-PB1(C) group. The LD_50_ of the two viruses reflected these differences: 13.6 PFU for rVN-PB1(U) and 23.7 PFU for rVN-PB1(C) (Fig. 4A and 4B). The infected mice showed significant decreases in body weight before death on days 5 and 6 after inoculation (*P*<0.05). Although infection with 1000 PFU was two high for mice to endure, differences of body weight were not significant (Fig. 4D). At an inoculation dose of 100 PFU, mice infected with rVN-PB1(U) virus started losing weight on day 2 and continued losing until they died. The rVN-PB1(C) virus caused delayed and milder decreases in weight from 5 d.p.i. to 8 d.p.i. (days post-infection). In this way, the weight loss of mice infected with rVN-PB1(U) and rVN-PB1(C) was found to differ significantly (*P*<0.05) (Fig. 4C). In summary, rVN-PB1 (U) was found to be more virulent to mice than rVN-PB1(C).

## Materials and Methods

### Ethics

All necessary permits were obtained for the animal experiments. The animal studies were carried out in strict accordance with the recommendations in the Guide for the Care and Use of Laboratory Animals of the National Institutes of Health and State Key Laboratory of Pathogens and Biosecurity of the Beijing Institute of Microbiology and Epidemiology and approved by the Institutional Animal Care and Use Committee of the AMMS under permit No. SCXK(Jun)-2007-004 (20130205). All experiments related to the influenza viruses were performed in approved biosafety level 3 (BSL-3) laboratories or BSL-3 animal facilities of Beijing Institute of Microbiology and Epidemiology of China (Permit Number: PBS013, 2013-05-20).

### Construction of plasmids

Eight plasmids containing recombinant A/Vietnam/1194/2004(VN1194) strain were produced for our previous study [19]. To rescue mutant virus on the basis of VN1194 strains with U and C at the 4^th^ position from the termini, information regarding primers was collected and shown in Table S1. The PCR procedure was performed using pfx polymerase (Promega), and the PCR products were digested with Bsa I (for PB2 and NA), and BsmB I (for PB1, PA, HA, NP, M, and NS). Then the digested products were ligated into pHW2000 plasmid digested with BsmB I, and the plasmids were sequenced to confirm correctness. Nine plasmids, called pHW-PB2-C_4_, pHW-PB1-U_4_, pHW-PB1-C_4_, pHW-PA-C_4_, pHW-HA-C_4_, pHW-NP-C_4_, pHW-NA-C_4_, pHW-M-C_4_, and pHW-NS-C_4_, were produced.

Two reporter plasmids, used to detect polymerase activity, were constructed according to the previous strategy [17]. The reporter plasmid expressing a negative-sense RNA transcript carrying the complete ORF of the Firefly luciferase gene flanked by untranslated regions of the NP segment. This plasmid has been described previously. These reporter plasmids were named as pHH-PB1_U_4_-LUC and pHH-PB1_C_4_-LUC.

### Cell culture

Human embryonic kidney 293 cells expressing SV40 Large T-antigen (293T) and Madin-Darby canine kidney cells (MDCK) were maintained in Dulbecco's modified Eagle's medium (DMEM, Gibico) with 10% FBS (fetal bovine serum, Gibco) and penicillin-streptomycin at 37°C with 5% CO_2_.

### Detection of polymerase transcriptional activity *in vitro*

Briefly, 293T cells were transfected with pHW2000 plasmid constructs encoding PB2, PB1, PA, and NP proteins and pHH21 plasmid construct expressing negative vRNA-like RNA (1 mg each) with lipofectamine 2000 (Invitrogen) according to the manufacturer's instructions. At 6 h post-transfection, the medium was replaced with DMEM with 10% FBS. Then cells were harvested for analysis at 24 h post-transfection. Then the Promega luciferase assay system was used to examine Firefly and *Renilla* luciferase activity. All experiments were independently repeated three times.

### Rescue of viruses with U_4_ and C_4_ mutations

Briefly, 293T cells were transfected with eight pHW2000 plasmids, pHW-PB2-C_4_, pHW-PA-C_4_, pHW-HA-C_4_, pHW-NP-C_4_, pHW-NA-C_4_, pHW-M-C_4_, pHW-NS-C_4_ and pHW-PB1-U_4_ or pHW-PB1-C_4_ (1 μg for each) with lipofectamine 2000 according to the manufacturer's instructions. The medium was replaced with DMEM containing 2% FBS at 6 h post-transfection and the supernatant was collected after 48 h. The stock virus was propagated in 11-day-old embryonated chicken eggs for 48 h. In order to identify the absence of unwanted mutation in 3′ end of viral genome, RNA were extracted, amplified and cloned into pEASY-Blunt Zero Cloning Vector (Transgen, China) to be sequenced. After the analysis of sequencing information, the recombinant viruses were named rVN-PB1(C) (all C_4_) and rVN-PB1(U) (only U_4_ in PB1).

### Measurement of RNA expression levels

MDCK cells in a 24-well plate were infected with reconstituted viruses at an MOI of 0.01 at 33°C, 37°C and 39°C for 1 h. After being washed three times with PBS, the cells were incubated with 1 ml of DMEM with 2% FBS at 33°C, 37°C and 39°C. The infected cells were collected at 2, 4, 6 and 8 hour post infection, then were lysed to extracted RNA with RNeasy Mini Kit (QIAGEN), stored at −80°C. Detection of RNA amount was applied with Tag-primed real-time RT-PCR assay which will be introduced later. All experiments were replicated in triplicate and means were obtained.

### Tag-primed real-time RT-PCR assay

To quantify the three types of influenza viral RNA (vRNA, cRNA, and mRNA) precisely and separately, the strand-specific real-time RT-PCR method was proposed[20]. This method is established on the basis of reverse transcription using tagged primers to add a tag sequence at the 5′ end and then real-time PCR using the tag portion as the forward primer and a segment-specific reverse primer ensured the specificity for quantifying the three types of RNA. The information of primers were covered in Table S2.

### Assessment of viral growth

MDCK cells in 24-well plates were infected with reconstituted viruses at an MOI of 0.01 at 33°C, 37°C, and 39°C for 1 h. They were washed three times with PBS and then incubated with 1 ml of DMEM with 2% FBS at 33°C, 37°C, and 39°C. The supernatants were harvested at 0, 12, 24, and 48 h after infection and stored at −80°C. Then titers were determined by a plaque formation assay in MDCK cells. All experiments were performed three times and the means were calculated.

### Survival time and weight loss in infected mice

Female Balb/C mice 4–6 weeks old weighing 14–17 g were purchased from the Beijing Experimental Animal Center and kept at 25°C and 40% humidity with rodent diet and water on top of cages in BSL-3 animal facilities. Before being subjected to the experiments, mice were anesthetized with 1% napental via intraperitoneal injection and randomly divided into 4 groups with equalized body weight among groups. Then, mice were inoculated with 50 μl of 10,000 PFU, 1000, 100, and 10 PFU of rVN-PB1(U) and rVN-PB1(C) by nasal dropping with Eppendorf pipette. Infected mice were monitored twice daily, and survival time, body weight, and general health were recorded. Then LD50 titers (50% mouse lethal dose) were calculated using the method described by Reed and Muench (1938). These titers are expressed as PFU.

### Euthanasia of mice

Infected mice were killed at humane end-points or at the predetermined end of the experiment. The humane end-points were strictly observed according to the scoring system based on the weight loss and on the symptom severity scale for influenza infection, which includes red eyes, ruffled fur, hunched back, altered breathing, and unresponsiveness. These symptoms have also been used in studies involving animal models of influenza infection [21]. Here, they were used to establish criteria for euthanasia: weight loss >25%  =  3 points; unresponsiveness  =  3 points; hunched back or altered breathing  =  2 points; red eye or ruffled fur  =  1 point. Animals were scored daily and each individual mouse with a score less than or equal to 3 points was humanely euthanized. All procedures, including inoculation and euthanasia, were performed under anesthesia to minimize the pain and suffering of infected animals. Analgesics were not used to prevent any influence on experimental outcomes.

### Statistical analysis

The *P* values indicating the significance of transcription activity were calculated using the Student's t test. The data for RNA expression levels, viral reproduction and weight loss were analyzed using a two-way ANAVA method, and the *P* values were calculated. All tests were performed using GraphPad Prism Software.

## Discussion

The increasing numbers of highly infectious, highly lethal influenza viruses, such as H5N1 and H7N9 avian influenza viruses, poses a serious threat to human [22]–[26]. In the last ten years, a large number of studies have shed light on the molecular foundation and pathogenesis of the highly pathogenic H5N1 virus. Various mutations within the coding regions of gene segments, such as PB2, PB1, HA, NA, and NS, were found to be associated with high levels of viral proliferation and strong cytokine stimulation [15], [27]–[29]. However, no study has yet focused on the association of UTRs with increased pathogenicity in the H5N1 influenza virus.

The variation, U_4_ or C_4_, was first observed by Robertson in 1979, which showed that the C_4_ was present in PB2, PB1 and PA gene segments, while U4 was present in other five gene segments for one strain of fowl plague virus [30]. The next year, Desselberger et al. found that PB2, PB1, PA, NA and M possessed C_4_, while the remaining genomic segments had U_4_ in A/PR/8/34 [5]. Furthermore, Lee and Seong suggested that U_4_ and C_4_ could temporally regulate viral transcription and replication with the help of two isogenic A/WSN/33 viruses with C_4_ or U_4_ in NA [10]. Moreover, Emmie de Wit et al. rescued the wild type, all C_4_ virus (C_4_ in eight segments), all U_4_ virus (U_4_ in eight segments) and series of virus in which each of the genomic segments was replaced with C_4_ for A/PR/8/34 and found that U_4_ and C_4_ did not affect virus production [31]. However, the mechanism underlining it had not been clear until Byong-Seok Choi' determined U_4_ and C_4_ RNA structures in NS using NMR spectroscopy and proposed the transcription-initiation models to explain the differential regulation of viral genomic synthesis [32]. Collectively, above-mentioned studies focused on the structure and function of U_4_ or C_4_ in non-polymerase gene segments, but it was not clear whether this variation in polymerase-related gene segments associates with genomic synthesis and even with viral pathogenicity. Therefore, in present study, we determined in details the association of U_4_/C_4_ variation in PB1 with viral pathogenicity.

The UTR sequences of genomic segments of A/Vietnam/1194/2004(H5N1) were not clear, and the VN1194 or other H5N1 virus was usually rescued with a cytosine at the fourth position in 3′ UTR of PB2, PB1 and PA, while with a uracil at this position of the other five segments [19], [33]. And our previous study showed that the rescued VN1194, i.e. rVN1194, with C_4_ at PB2, PB1 and PA, while U_4_ at the other five segments was as high pathogenic as the wild VN1194 virus, with an MLD_50_ less than 1 PFU [19], [33]. Because the difference in pathogenicity contributed by the U4/C4 variation in PB1 was not large enough to be identified in the background of rVN1194 (data not shown), we re-rescued the VN1194 virus with C_4_ in all segments, which was named as rVN-PB1(C). The rVN-PB1(C) and the rVN-PB1(U), in which C4 in all segments except PB1 (U4) were less pathogenic to mice than the rVN1194, with LD50 of more than 10 PFU. Though rVN-PB1(C) and rVN-PB1(U) were artificially rescued and different from the wild VN1194 virus, the difference in RdRp activity, virus replication and pathogenicity in mice is definitely caused by the U_4_/C_4_ variation. And we determined the difference caused by the variation in details with rVN-PB1(C) and rVN-PB1(U).

Firstly, the higher transcriptional activity of PB1(U_4_) was confirmed by a dual luciferase reporter system at all three various temperatures, i.e., 33°C, 37°C, and 39°C (Fig. 3). Furthermore, differences of transcription activity attributable to polymerase binding affinity have been found [18]. Taken together, these results suggest that U_4_ and C_4_ can differentially regulate their RdRp binding and recognition affinity.

Our results of RNA expression level showed that, the similar patters could be found at all three temperatures: U_4_ could up-regulate mRNA synthesis and temporally down-regulate cRNA synthesis (Fig.2), which agreed with the transcription-initiation models for U_4_ and C_4_ promoters proposed by Lee et al. [32]. Maybe the U_4_ formed a more stable structure than C4 did, initialed higher transcription acitivity, and delayed cRNA synthesis at the same time. However, the mechanism under the changes of vRNA amounts, the vRNA levels of U_4_ promoter are much less than that of C_4_ promoter, was not clear. There might be one possible explanation: the stable structural of U_4_ at 3′ end of vRNA also means the stable structural of A_4_ at 5′ end of cRNA, which may delay the synthesis of new vRNA from cRNA. Taken together, acquisition of U_4_ associated with differential regulation of mRNA, cRNA, and vRNA synthesis.

Then, the effects of this variation in viral reproduction were determined. Repeated experiments at different temperatures demonstrated that U_4_ contributed to a higher virus replication in MDCK cells (Fig. 3). In the early stage of infection, the titers of two virus did not show significant difference at 33°C and 37°C. However, single U_4_ virus replicated more efficiently after 12 or 24 h.p.i. (Fig.3A and 3B), and the virus growth was affected simply by substitution of this variation in PB1.

Finally, the contribution of U_4_ or C_4_ in PB1 to pathogenicity was assessed in mice. Their pathogenicity agree with their replication efficiency: virus with higher replication efficiency showed higher mortality and morbidity. Although rVN-PB1(U) and rVN-PB1(C) are both highly pathogenic in mice, a significant difference in weight loss was observed between the two viruses: rVN-PB1(U) is more pathogenic (Fig. 4). The present study is the first to show variations in the UTRs of H5N1 influenza virus to be associated with large amounts of viral pathogenicity.

In summary, the variation of U_4_ or C_4_ at the 3′ end of UTRs in PB1 segment of H5N1 influenza virus promotes changes in RdRp activity, RNA expression level viral proliferation and pathogenicity. And we also concerned that: 1) sequencing and uploading of accurate UTR information, especially determining the variation of this nucleotide at 4^th^ position from 3′end of vRNA, are necessary; 2) defining the substitution of U_4_ or C_4_ should be involved in the monitoring outbreak of new flu and transmission of avian influenza virus to human.

## Supporting Information

Table S1 **Primers for construction of reporter system.**
(DOCX)

Table S2 **Primers for RT-PCR and real-time PCR.**
(DOCX)
