# Supplementary material for: U4 at the 3′ UTR of PB1 Segment of H5N1 Influenza Virus Promotes RNA Polymerase Activity and Contributes to Viral Pathogenicity
Source: PLoS One. 2014 Mar 27;9(3):e93366. doi: 10.1371/journal.pone.0093366 (PMC3968160; doi:10.1371/journal.pone.0093366)
Supplement: Table S1 — Primers for construction of reporter system. (DOCX) [file pone.0093366.s001.docx]

**Table S1. Primers for construction of reporter system**

| Purpose | Primer name | Sequence(5′–3′) |
| --- | --- | --- |
| Construction | PF(U4) | TATTGGTCTCAGGGAGC**A**AAAGCAG |
|  | PF(C4) | TATTGGTCTCAGGGAGC**G**AAAGCAG |
|  | PR | ATATGGTCTCGTATTAGTAGAAACAAGG |
