# Supplementary material for: U4 at the 3′ UTR of PB1 Segment of H5N1 Influenza Virus Promotes RNA Polymerase Activity and Contributes to Viral Pathogenicity
Source: PLoS One. 2014 Mar 27;9(3):e93366. doi: 10.1371/journal.pone.0093366 (PMC3968160; doi:10.1371/journal.pone.0093366)
Supplement: Table S2 — Primers for RT-PCR and real-time PCR. (DOCX) [file pone.0093366.s002.docx]

**Table S2. Primers for RT-PCR and real-time PCR**

| Purpose | Target | Primer name | Sequence(5’-3’) |
| --- | --- | --- | --- |
| Reverse transcription | PB1-mRNA | Tagged4PB1-mRNA | CCAGATCGTTCGAGTCGTTTTTTTTTTTTTTTTTCACGAGGGACAAGCT |
|  | PB1-cRNA | Tagged4PB1-cRNA | GCTAGCTTCAGCTAGGCATC AGTAGAAACAAGGCATTTTT |
|  | PB1-vRNA | Tagged4PB1-vRNA | GGCCGTCATGGTGGCGAATCATTGGTGTTACGGTTAT |
| Real-time PCR | PB1-mRNA | Tag-mRNA | CCAGATCGTTCGAGTCGT |
|  | PB1-cRNA | Tag-cRNA | GCTAGCTTCAGCTAGGCATC |
|  | PB1-vRNA | Tag-vRNA | GGCCGTCATGGTGGCGAAT |
|  | PB1-mRNA | PB1-mRNA | AAGAGTTTGCTGAGATCATG |
|  | PB1-cRNA | PB1-cRNA | AAGAGTTTGCTGAGATCATG |
|  | PB1-vRNA | PB1-vRNA | ATCGGTATGTGTATCTGT |
|  | GAPDH | PF-GAPDH | GACTCATGACCACAGTCCATGC |
|  |  | PR-GAPDH | AGAGGCAGGGATGATGTTCTG |

The underlined sequences are added tags
